# Quality of care and maternal mortality in a tertiary-level hospital in Mozambique: a retrospective study of clinicopathological discrepancies

**DOI:** 10.1016/S2214-109X(20)30236-9

**Published:** 2020-06-17

**Authors:** Clara Menéndez, Llorenç Quintó, Paola Castillo, Fabiola Fernandes, Carla Carrilho, Mamudo R Ismail, Cesaltina Lorenzoni, Juan Carlos Hurtado, Natalia Rakislova, Khátia Munguambe, Cinta Moraleda, Maria Maixenchs, Eusebio Macete, Inacio Mandomando, Miguel J Martínez, Pedro L Alonso, Quique Bassat, Jaume Ordi

**Affiliations:** a ISGlobal, Hospital Clinic—Universitat de Barcelona, Barcelona, Spain; b Department of Pathology, Hospital Clinic—Universitat de Barcelona, Barcelona, Spain; c Department of Microbiology, Hospital Clinic—Universitat de Barcelona, Barcelona, Spain; d Centro de Investigação em Saúde de Manhiça, Maputo, Mozambique; e Consorcio de Investigación Biomédica en Red de Epidemiología y Salud Pública (CIBERESP), Madrid, Spain; f Department of Pathology, Maputo Central Hospital, Maputo, Mozambique; g Faculty of Medicine, Eduardo Mondlane University, Maputo, Mozambique; h Catalan Institution for Research and Advanced Studies, Barcelona, Spain; i Pediatric Infectious Diseases Unit, Pediatrics Department, Hospital Sant Joan de Déu, University of Barcelona, Barcelona, Spain

## Abstract

**Background:**

Although an increasing number of pregnant women in resource-limited areas deliver in health-care facilities, maternal mortality remains high in these settings. Inadequate diagnosis and management of common life-threatening conditions is an important determinant of maternal mortality. We analysed the clinicopathological discrepancies in a series of maternal deaths from Mozambique and assessed changes over 10 years in the diagnostic process. We aimed to provide data on clinical diagnostic accuracy to be used for improving quality of care and reducing maternal mortality.

**Methods:**

We did a retrospective analysis of clinicopathological discrepancies in 91 maternal deaths occurring from Nov 1, 2013, to March 31, 2015 (17 month-long period), at a tertiary-level hospital in Mozambique, using complete diagnostic autopsies as the gold standard to ascertain cause of death. We estimated the performance of the clinical diagnosis and classified clinicopathological discrepancies as major and minor errors. We compared the findings of this analysis with those of a similar study done in the same setting 10 years earlier.

**Findings:**

We identified a clinicopathological discrepancy in 35 (38%) of 91 women. All diagnostic errors observed were classified as major discrepancies. The sensitivity of the clinical diagnosis for puerperal infections was 17% and the positive predictive value was 50%. The sensitivity for non-obstetric infections was 48%. The sensitivity for eclampsia was 100% but the positive predictive value was 33%. Over the 10-year period, the performance of clinical diagnosis did not improve, and worsened for some diagnoses, such as puerperal infection.

**Interpretation:**

Decreasing maternal mortality requires improvement of the pre-mortem diagnostic process and avoidance of clinical errors by refining clinical skills and increasing the availability and quality of diagnostic tests. Comparison of post-mortem information with clinical diagnosis will help monitor the reduction of clinical errors and thus improve the quality of care.

**Funding:**

Bill & Melinda Gates Foundation and Instituto de Salud Carlos III.

## Introduction

The increasing number of pregnant women delivering in health facilities in low-income and middle-income countries (LMICs; 58% in 1990 and 78·3% in 2016)^1^ has not resulted in the expected reduction in maternal mortality. More than 300 000 women die annually during childbirth, with 99% of these deaths disproportionally occurring in LMICs. Such high mortality could have many causes, including delays in the decision to seek care, arrival at a health facility, and provision of adequate care.^2^ Importantly, delays in the provision of adequate care include inadequacies in the quality of care provided by health services, since giving birth in a health facility does not necessarily imply a safe birth in many parts of the world. A key factor not sufficiently recognised that leads to provision of poor quality care to pregnant women in health facilities is imprecise diagnosis of the illnesses that led to death.

Inaccurate knowledge of the cause of death hampers adequate evaluation of the quality of clinical diagnosis and management, hindering reduction of clinical errors. Clinical diagnoses should be compared against complete diagnostic autopsy, the gold standard for ascertainment of cause of death, to determine the frequency and magnitude of clinical errors.^3^, ^4^ Historically, comparative analysis of clinicopathological discrepancies has shown that clinical errors are not uncommon, even in hospitals in high-income countries.^5^, ^6^, ^7^ In sub-Saharan Africa, where access to diagnostic tools is restricted and infectious diseases are extremely prevalent, the rate of clinicopathological discrepancies is very high.^8^, ^9^ For maternal deaths in LMICs, data on clinicopathological discrepancies are limited to two studies from Nigeria and Mozambique, reporting either a low^10^ or high^11^ frequency of clinical errors.^10^, ^11^

We analysed the clinicopathological discrepancies in a series of maternal deaths from Mozambique and assessed changes over 10 years in the diagnostic process. We aimed to provide data on clinical diagnostic accuracy to be used for improving quality of care and reducing maternal mortality.

**Research in context**

**Evidence before this study**

Clinicians can only diagnose diseases they have considered in the differential diagnostic process and for which they have been looking. Resource-poor settings often do not have adequate diagnostic tools and skilled medical staff. In these settings, clinicopathological correlation can help improve clinical diagnostic performance by providing fundamental information on the specific diseases that are mostly frequently misdiagnosed. We searched PubMed for studies published in English that explored clinical errors in low-income countries between Jan 15, 2003, and Feb 15, 2018, using the search terms (“concordance autopsy and clinical diagnosis”, “clinico-pathological errors” and “clinico-pathological discrepancies”) combined with the term “maternal deaths”. We identified three studies, two of which were done in low-income countries (Nigeria and Mozambique). The Nigerian study reported a low frequency of clinical errors (10%). By contrast, the study in Mozambique found clinical errors were more frequent (40%).

**Added value of this study**

We present a retrospective analysis of clinicopathological discrepancies in 91 maternal deaths occurring from Nov 1, 2013, to March 31, 2015 (a 17 month period), at a tertiary-level hospital in Mozambique, using complete diagnostic autopsy. We estimated the performance of clinical diagnosis and classified clinicopathological discrepancies as major and minor errors. We also had the unique opportunity to compare the results of this analysis with those of a similar study done in the same setting 10 years earlier. Our findings show that a major clinical diagnostic error was identified in almost 40% of patients, and clinical diagnosis had low sensitivity for both puerperal and non-obstetric infections. In the case of eclampsia, although the sensitivity of the clinical diagnosis was 100%, the positive predictive value was only 33%, indicating that the probability a women clinically diagnosed with eclampsia died of this condition was fairly low.

**Implications of all the available evidence**

Reduction in maternal mortality in low-income settings requires an effort to improve the diagnostic process of maternal illness and avoid clinical errors by refining clinical skills and increasing the availability and quality of diagnostic tests. The comparison of post-mortem information (either complete diagnostic autopsies or minimally invasive autopsy methods) with clinical diagnosis might be useful to monitor the reduction of clinical errors and thus improve the quality of care and maternal health.

## Methods

### Study area and design

This retrospective study was done at the Maputo Central Hospital (Maputo, Mozambique), a 1500-bed government-funded tertiary-level health-care facility. Recruitment of maternal deaths was done from Nov 1, 2013, to March 31, 2015 (17-month period). All deceased women who fulfilled the standard WHO definition of a pregnancy-related death,^12^ and for whom the family had given verbal informed consent for the autopsy requested by the clinician, were included. Accidental or incidental deaths were excluded. Following the guidelines of the Ministry of Health of Mozambique, all maternal deaths occurring at the Maputo Central Hospital undergo a complete diagnostic autopsy unless the family does not provide consent.

This study received approval from the National Bioethics Committee of Mozambique (342/CNBS/13) and the Clinical Research Ethics Committee of the Hospital Clinic of Barcelona (Spain; 2013/8677).

### Procedures

A complete dissection was done with macroscopic evaluation of all organs according to a standardised protocol.^13^ Samples of grossly identified lesions and of solid organs, including the uterus, were collected for histological examination; additionally, samples of blood and cerebrospinal fluid were obtained. When available, the placenta was macroscopically evaluated and sampled.

Histological evaluation comprised staining with haematoxylin and eosin in all samples and additional histochemical or immunohistochemical stains (eg, Ziehl-Neelsen or *Plasmodium falciparum* immunohistochemical staining) when needed. The extensive microbiological analysis done has been reported in detail elsewhere.^14^ Briefly, universal screening was done, which comprised detection of *P falciparum* by PCR, detection of antibodies against HIV-1 and HIV-2 and HIV viral load, and bacterial or fungal cultures of blood and cerebrospinal fluid. Additional microbiological screening was applied to HIV-positive cases, including real time PCR in cerebrospinal fluid for *Toxoplasma gondii, Mycobacterium tuberculosis*, and *Cryptococcus* spp and real-time PCR in lung samples for *Pneumocystis jirovecii*. Molecular methods were used in cases in which the histological features were discordant with the culture results (eg, pneumonia by histology and no infectious agent identified on culture).

Patient data, including demographic information, previous medical history, and inpatient admission process (collected by clinicians in charge, including obstetricians) were extracted from medical records and recorded in a standardised questionnaire by a study medical doctor (QB). Up to five clinical diagnoses registered in medical records by the caring clinicians were selected and abstracted. The first diagnosis listed was regarded as the main diagnosis, and the remaining diagnoses were classified as secondary.

Macroscopic, microscopic, and microbiological findings of complete diagnostic autopsies and any available clinical information were evaluated by a panel of multidisciplinary experts that comprised clinical (maternal and child health) and laboratory (pathology and microbiology) specialists, and the final complete diagnostic autopsy diagnosis was assigned. As previously described,^14^ all morbid conditions directly leading to death, any underlying conditions, and any other clinically significant conditions possibly contributing to death were classified as either direct obstetric or indirect obstetric deaths, and codified according to the International Classification of Diseases, 10th revision.^12^, ^15^ Diseases were grouped into the following eight categories: (1) pregnancies with abortive outcome; (2) hypertensive disorders in pregnancy, childbirth, and puerperium; (3) obstetric haemorrhage; (4) pregnancy-related infections; (5) other obstetric complications; (6) unanticipated complications of management; (7) non-obstetric complications; and (8) unexplained deaths. We considered categories 1 to 6 direct obstetric deaths, whereas category 7 was considered to correspond to indirect obstetric deaths. When more than one severe diagnosis was identified, the disease most likely to have caused the death was considered the final complete diagnostic autopsy diagnosis.^14^

Diagnostic discrepancies were classified as major or minor.^16^, ^17^ Major discrepancies involved major diagnoses and were classified as class I or class II. Class I refers to discrepancies in which the knowledge of the correct diagnosis before death would have led to changes in clinical management that could have prolonged survival or cured the patient (eg, pyogenic meningitis treated as eclampsia). In class II errors, patient survival would have not been modified (eg, fulminant hepatitis treated as sepsis). Minor discrepancies involved minor diagnoses and were classified as class III (non-diagnosed diseases with symptoms that should have been treated—eg, mild aspiration pneumonia in a patient with eclampsia) and class IV (non-diagnosed diseases with possible epidemiological or genetic importance—eg, schistosomal infections). Correctly diagnosed patients were classified as class V. Class VI comprised non-classifiable cases (autopsy unsatisfactory or with no clear diagnosis).

For analysis of clinicopathological discrepancies, two masked investigators assessed each case; their evaluations were compared and a third rater evaluated any discrepant cases. The following information was provided to each rater: autopsy final diagnosis, antecedent causes, and other significant conditions and clinical diagnoses (main diagnosis, and up to a maximum of four additional diagnoses) extracted from the medical record. Clinicopathological correlation was determined by assessing whether the complete diagnostic autopsy diagnosis was identified among any of the clinical diagnoses. A case was considered discrepant when there was no coincidence between any of the five clinical diagnoses listed by the clinician and the final cause of death identified in the complete diagnostic autopsy. In each case, only the worst diagnostic error was considered.

We did a comparative analysis of the performance of the clinical diagnosis of four main maternal death categories between the current findings and those of a study undertaken 10 years earlier in the same hospital and using the same methods to determine cause of death.^11^

### Statistical analysis

We assessed concordance between raters with the κ statistic.^18^ We compared proportions by χ^2^ test and used logistic regression with penalised likelihood to evaluate factors associated with major clinical errors.^19^, ^20^ We used penalised likelihood to mitigate the bias caused by rare events in the dataset, as major errors were infrequent or non-existent for some covariates included in the analysis of associations or a combination of them in multivariable analyses (eg, ectopic gravidity, bloody diarrhoea, and choluria). This situation is referred to as separation or monotone likelihood and produces infinite estimates for some coefficients. In such a situation, it can be useful to maximise Firth's penalised likelihood, rather than the usual likelihood.^19^

We calculated the sensitivity, specificity, positive predictive value, and negative predictive value for each diagnosis. We defined false-negative diagnoses as discrepancies for which the autopsy diagnosis was in the assessed diagnostic category, but the clinical diagnosis was in another diagnostic category. We defined false-positive diagnoses as discrepancies for which the clinical diagnosis was in the diagnostic category but not the autopsy diagnosis. We estimated a multivariable adjusted model using all covariates with p≤0·15 in the crude analysis.

Data were analysed with STATA (version 15).

### Role of the funding source

The funders of the study had no role in study design, data collection, data analysis, data interpretation, or writing of the report. The corresponding author had full access to all the data in the study and had final responsibility for the decision to submit for publication.

## Results

Of 136 maternal deaths that occurred at Maputo Central Hospital during the 17-month study period, 91 (67%) (median age 28 years, range 15–39) were included in the study. At the time of death, 20 (22%) of 91 women were pregnant, three (3%) died during delivery (one spontaneous miscarriage or stillbirth), and 68 (75%) died during the puerperium (ten after spontaneous miscarriage or stillbirth and one ectopic pregnancy). The mean time between hospital admission and death was 108·7 h (SD 175·0). 16 (18%) of 91 women were primigravidae, 74 (81%) were multigravidae, and in one (1%) woman parity was unknown. 63 (69%) of 91 women lived in an urban area, 26 (29%) in a rural area, and in two (2%) women the place of residence was unknown.

**Figure 1:**
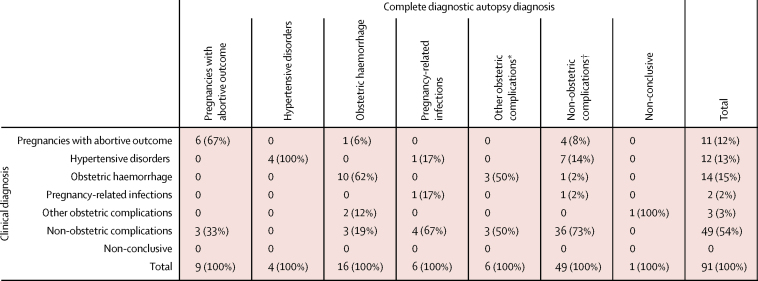
Distribution of the diagnostic groups of causes of maternal death according to clinical diagnosis and the diagnosis by complete diagnostic autopsy in the 91 maternal deaths included in the study *One case of cardiomyopathy in the puerperium, three cases of complication of labour and delivery, unspecified, and two cases of disruption of caesarean section wound. † 33 (67%) of 49 non-obstetric complications were infectious diseases: four cases of bacterial sepsis, 12 cases of pneumonia, two cases of meningitis, ten cases of HIV or AIDS-related infections, four cases of malaria, and one case of pyelonephritis. κ statistic 0·4353 (p<0·0001, moderate agreement).

In 41 (45%) of 91 patients, the cause of death attributed by the complete diagnostic autopsy was a direct obstetric complication, which included complications of abortion (nine [10%] of 91 women), hypertensive disorders (four [4%] women), obstetric haemorrhage (16 [18%] women), pregnancy-related infections (six [7%] women), and other obstetric complications (six [7%] women). In 49 (54%) of 91 women, the cause of death attributed by the complete diagnostic autopsy was an indirect obstetric disease. Most non-obstetric complications were infections (33 of 49 women: 12 pneumonia cases, ten HIV-related infections [four cryptococcosis, four tuberculosis, and two pneumonia cases, caused by *Staphylococcus aureus* and *Streptococcus pneumoniae*], four severe malaria cases, four disseminated infections [bacterial sepsis] two meningitis cases, and one pyelonephritis case). In one case the autopsy did not yield a conclusive diagnosis (figure 1).

The clinical diagnosis and complete diagnostic autopsy diagnosis agreed in 57 (63%) of 91 cases, with a κ statistic of 0·4353 (p<0·0001; moderate agreement). For clinical diagnosis compared with complete diagnostic autopsy diagnosis, the sensitivity for hypertensive disorders was 100% but the positive predictive value was 33% (table 1). For pregnancy-related infections, the sensitivity was low (17%) with a low positive predictive value (50%). Although the sensitivity for non-obstetric complications was 73%, it was only 48% for the 33 cases of non-obstetric infections (data not shown).

**Table 1:** Performance of clinical diagnosis compared with the diagnosis of the complete diagnostic autopsy by category of cause of death

	**n**	**True positives**	**True negatives**	**False positives**	**False negatives**	**Sensitivity**	**Specificity**	**Positive predictive value**	**Negative predictive value**
Pregnancies with abortive outcome	9	6	77	5	3	67%	94%	55%	96%
Hypertensive disorders	4	4	79	8	0	100%	91%	33%	100%
Obstetric haemorrhage	16	10	71	4	6	62%	95%	71%	92%
Pregnancy-related infections	6	1	84	1	5	17%	99%	50%	94%
Other obstetric complications	6	0	82	3	6	0	96%	0	93%
Non-obstetric complications	49	36	29	13	13	73%	69%	73%	69%
Non-conclusive	1	0	90	0	1	0	100%	NA	99%

NA=not applicable.

We identified a clinicopathological discrepancy in 35 (38%) of 91 cases. All diagnostic errors observed were classified as major discrepancies. 30 were classified as class I and five as class II major errors. In 55 (60%) maternal deaths, there was complete agreement between the clinical and the autopsy diagnoses (class V). One case was classified as class VI (non-classifiable). The percentage of diagnostic errors for each group is shown in table 2.

**Table 2:** Distribution of clinical errors by diagnostic group of cause of death

	**Class I**	**Class II**	**Class III**	**Class IV**	**Class V**	**Class VI**	**Total**
Pregnancies with abortive outcome	0	0	0	0	9 (100%)	0	9 (100%)
Hypertensive disorders	0	0	0	0	4 (100%)	0	4 (100%)
Obstetric haemorrhage	0	0	0	0	16 (100%)	0	16 (100%)
Pregnancy-related infections	4 (67%)	1 (17%)	0	0	1 (17%)	0	6 (100%)
Other obstetric complications	0	0	0	0	6 (100%)	0	6 (100%)
Non-obstetric complications	26 (53%)	4 (8%)	0	0	19 (39%)	0	49 (100%)
Non-conclusive	0	0	0	0	0	1 (100%)	1 (100%)
Total	30 (33%)	5 (5%)	0	0	55 (60%)	1 (1%)	91 (100%)

In 70 (77%) of 91 cases, the two raters attributed the same type of error. The κ score between the two independent evaluators was 0·5639 (p<0·0001; moderate agreement). The autopsy diagnosis and the first two clinical diagnoses for each case classified as major (type I or II errors) are shown in the appendix (p 1).

We did logistic regression analysis of the factors potentially associated with the occurrence of clinical diagnostic errors (table 3). A history of medical treatment before admission, a low coma score, and history of fever or current fever were associated with an increased risk of clinical errors in the crude analysis. However, vaginal bleeding was associated with decreased odds that the clinical diagnosis was discrepant with a major error. The significance of these associations was not maintained in an adjusted analysis (table 3).

**Table 3:** Crude and adjusted analysis of factors associated with major diagnostic errors

		**Type of error**		**Crude analysis**		**Adjusted analysis**	
		None or minor*	Major	OR (95% CI)	p value†	OR (95%CI)	p value†
**Case characteristics**							
Status of the patient					0·1217		0·4110
	Pregnant	11 (20%)	14 (40%)	1 (ref)	..	1 (ref)	..
	Delivery or abortion	15 (27%)	7 (20%)	0·38 (0·12–1·23)	..	0·67 (0·08–5·26)	..
	Postpartum	30 (54%)	14 (40%)	0·38 (0·14–1·02)	..	0·35 (0·08–1·67)	..
**Anamnesis at admission**							
Vaginal bleeding					0·0382		0·2979
	No	26 (46%)	23 (66%)	1 (ref)	..	1 (ref)	..
	Yes	30 (54%)	10 (29%)	0·39 (0·16–0·95)	..	0·40 (0·07–2·26)	..
	Unknown	0	2 (6%)	..	..	..	..
Hypertension					0·8547		..
	No	25 (45%)	20 (57%)	1 (ref)	..	..	..
	Yes	1 (2%)	1 (3%)	1·24 (0·12–12·87)	..	..	..
	Unknown	30 (54%)	14 (40%)	..	..	..	..
Pre-admission medication					0·0414		0·0648
	No	34 (61%)	14 (40%)	1 (ref)	..	1 (ref)	..
	Yes	18 (32%)	19 (54%)	2·51 (1·04–6·07)	..	3·25 (0·93–11·35)	..
	Unknown	4 (7%)	2 (6%)	..	..	..	..
Axillary temperature		37·03 (1·30) [33]	37·49 (1·25) [23]	1·31 (0·86–1·98)	0·2057	..	..
**Nutritional status**					**0·8098**		**..**
	Normal	29 (52%)	18 (51%)	1 (ref)	..	..	..
	Cachexia or malnutrition	2 (4%)	1 (3%)	0·96 (0·12–7·86)	..	..	..
	Obesity	4 (7%)	4 (11%)	1·59 (0·38–6·66)	..	..	..
	Unknown	21 (38%)	12 (34%)	..	..	..	..
Oedemas					0·6619		..
	No	42 (75%)	27 (77%)	1 (ref)	..	..	..
	Yes	14 (25%)	7 (20%)	0·80 (0·29–2·18)	..	..	..
	Unknown	0	1 (3%)	..	..	..	..
Pallor					0·7733		..
	No	23 (41%)	15 (43%)	1 (ref)	..	..	..
	Yes	33 (59%)	19 (54%)	0·88 (0·38–2·07)	..	..	..
	Unknown	0	1 (3%)	..	..	..	..
**Neurological exam**							
General status					0·1174		..
	Conscious	37 (66%)	14 (40%)	1 (ref)		2·74 (0·09–85·22)	..
	Lethargy	2 (4%)	3 (9%)	3·62 (0·64–20·47)		1·69 (0·18–15·81)	..
	Confusion or agitation	7 (12%)	8 (23%)	2·93 (0·92–9·29)		1·98 (0·03–123·96)	..
	Unconscious	10 (18%)	10 (29%)	2·59 (0·91–7·38)		2·74 (0·09–85·22)	..
Glasgow coma scale		13·54 (3·42) [50]	11·61 (3·84) [33]	0·87 (0·77–0·98)	0·0272	0·89 (0·57–1·40)	0·6098
Stiff neck or meningeal syndrome					0·5886		..
	No	53 (95%)	53 (95%)	1 (ref)	..	..	..
	Yes	2 (4%)	2 (4%)	1·65 (0·27–10·02)	..	..	..
	Unknown	1 (2%)	1 (2%)	..	..	..	..
**Obstetric history**							
Parity					0·2647		..
	Primigravidae	8 (14%)	8 (23%)	1 (ref)	..	..	..
	Multigravidae	48 (86%)	26 (74%)	0·55 (0·19–1·58)	..	..	..
	Unknown	0	1 (3%)	..	..	..	..
Gestational age (weeks)		29·06 (10·62) [48]	26·83 (8·98) [30]	0·98 (0·94–1·02)	0·3405	..	..
**Fever during pregnancy**					**0·0554**		**0·6953**
	No	32 (57%)	12 (34%)	1 (ref)	..	1 (ref)	..
	Yes	14 (25%)	14 (40%)	2·60 (0·98–6·91)	..	0·65 (0·07–5·74)	..
	Unknown	10 (18%)	9 (26%)	..	..	..	..
Malaria during pregnancy					0·8330		..
	No	24 (43%)	14 (40%)	1 (ref)	..	..	..
	Yes	3 (5%)	2 (6%)	1·21 (0·21–6·93)	..	..	..
	Unknown	29 (52%)	19 (54%)	..	..	..	..
Anaemia					0·1117		0·7062
	No	14 (25%)	15 (43%)	1 (ref)	..	1 (ref)	..
	Yes	34 (61%)	17 (49%)	0·47 (0·19–1·19)	..	1·34 (0·29–6·24)	..
	Unknown	8 (14%)	3 (9%)	..	..	..	..
**Clinical course**							
Fever during admission					0·0214		0·4492
	No	35 (62%)	12 (34%)	1 (ref)	..	1 (ref)	..
	Yes	20 (36%)	20 (57%)	2·84 (1·17–6·91)	..	2·09 (0·31–14·01)	..
	Unknown	1 (2%)	3 (9%)	..	..	..	..
HIV status					0·4325		..
	Negative	4 (7%)	6 (17%)	1 (ref)	..	..	..
	Positive	26 (46%)	22 (63%)	0·59 (0·16–2·22)	..	..	..
	Unknown	26 (46%)	7 (20%)	..	..	..	..
Lower haemoglobin		7·15 (2·49) [42]	7·87 (2·68) [24]	1·11 (0·92–1·35)	0·2787	..	..
Malaria rapid test					0·1598		..
	Negative	19 (34%)	18 (51%)	1 (ref)	..	..	..
	Positive	4 (7%)	0 (0%)	0·12 (0·01–2·33)	..	..	..
	Unknown	33 (59%)	17 (49%)	..	..	..	..

Data are n (%) or mean (SD) [n], unless otherwise indicated. OR=odds ratio.

* There were no minor clinical errors identified for case characteristics, anamnesis at admission, or neurological exam.

† Penalised logistic regression.

**Figure 2:**
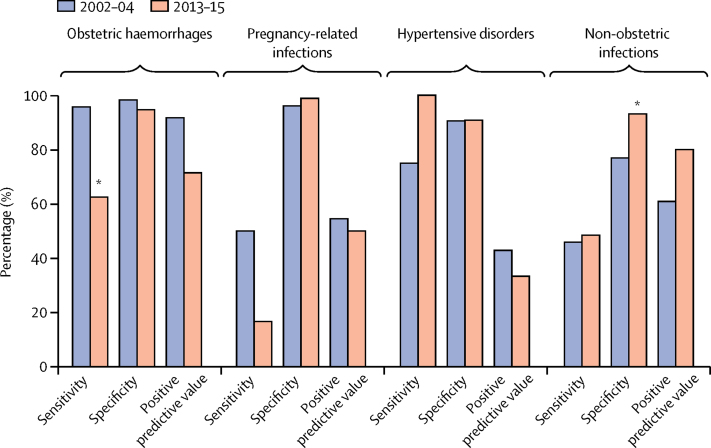
Comparison of the performance of the diagnostic process in maternal deaths over time *p<0·05 from Fisher's exact test for difference in proportions.

Characteristics of the patients (pregnancy status, parity, age, and residence) were similar in the present study and the previous study in the same setting (data not shown).^11^ The frequency of major diagnostic errors (class I and II) was similar between the 2002–04 and 2013–15 periods (40% and 38%, respectively). Over the 10-year comparison, the sensitivity decreased for obstetric haemorrhages from 96% to 62% (p=0·0127). The sensitivity for eclampsia increased from 75% to 100% (p=0·5286), but the positive predictive value decreased from 43% to 33% (p=0·7189; lower probability of the clinical diagnosis being correct). For non-obstetric infections, specificity increased; sensitivity remained lower than 50%, with high a proportion of false-negative diagnosis (figure 2).

## Discussion

In this study, we identified a clinicopathological discrepancy in 35 (38%) of 91 women. All diagnostic errors observed were classified as major discrepancies, implying that a change in clinical management could have substantially modified prognosis and potentially averted death. The proportion of discrepancies observed was similar to that in a study in the same setting more than 10 years earlier,^11^ suggesting that, although improvements in clinical management might have been introduced, these have not translated into a substantial reduction in diagnostic errors. Importantly, such a proportion of clinical errors is larger than that observed in a Nigerian study (22 of 230 cases), highlighting that even in resource-constrained settings it is possible to achieve better premortem diagnosis.^10^

In eight of 12 patients clinically diagnosed with eclampsia, the condition was not confirmed in the autopsy, resulting in the lowest positive predictive value. We also observed a high number of false negative diagnoses for infectious diseases, with some of these cases clinically diagnosed with eclampsia as the cause of death. Thus, physicians tended to overdiagnose eclampsia as a cause of maternal mortality. Even if improvements in clinical cognition and management of frequent obstetric complications were introduced, misdiagnosis of eclampsia might have led to death because of insufficient provision of specific treatment for the actual condition. Obstetric infections had the lowest sensitivity, with only one case diagnosed clinically as an obstetric infection of the six cases identified as such by complete diagnostic autopsy. Infectious diseases tend to be overlooked as a cause of maternal mortality, and clinicians should proactively screen for infections, particularly in the presence of fever or a history of fever.^21^

Among the variables considered to possibly influence the clinical diagnosis, no variable was independently associated with a higher or lower probability of a clinical error. Larger sample sizes are probably required to evaluate these associations.

When comparing the current study with the previous study in the same setting,^11^ neither the rate of maternal autopsies (high) nor the conditions to request them (all cases without selection) changed, which are necessary conditions for a valid comparison of the clinical diagnostic performance over time. The overall performance of the clinical diagnosis in the main diagnostic groups did not change over time. Overall, these findings indicate that improvements in clinical recognition of the investigated diseases have not occurred and use of diagnostic tests has not increased during this period. Two retrospective analyses on diagnostic errors during three consecutive decades from a high-income country showed a significant reduction in major clinical errors over time, explained by improvements in clinical skills and by use of more sensitive and specific diagnostic procedures.^22^ Reasons for the findings over time in the current study are difficult to confirm but it is likely that improvements in clinical skills and new diagnostic tools—if introduced—have not been sufficient or adequate to reduce the most critical clinical errors. Improvements in medical performance to reduce false negative diagnoses and more specific diagnostic tests to reduce false positive diagnoses are urgently needed to reduce maternal mortality. In this respect, proactive screening among sick pregnant women (prepartum or postpartum) for life-threatening infections, such as malaria, tuberculosis, or bacterial pneumonia or meningitis, particularly in the context of a history of fever or loss of consciousness, would appear to be a potentially immediate quick win in this setting. Additionally, making post-mortem data available to clinicians so that clinicopathological discrepancies can serve as a vehicle for ongoing diagnostic improvement should be organised. The constitution in Maputo Central Hospital of a maternal mortality committee, comprising clinicians and pathologists, that critically reviews all available information regarding maternal deaths is a step in the right direction.

The main limitation of this study is that it was done in a referral hospital, thus extrapolation of the findings to smaller or rural hospitals might not be possible. The rate of clinical errors is lower in larger hospitals^23^ but the number of complicated pregnancies, which have an increased diagnostic difficulty, tends to be higher in large hospitals. However, complete diagnostic autopsy is not feasible in smaller or rural health facilities because of insufficient personnel and resources. Another possible limitation of the study is that, although we met three of the four conditions proposed for complete diagnostic autopsy to monitor clinical diagnosis performance,^24^ we did not assess the error of the autopsy itself, which would have required a specific study. Finally, a possible limitation could be the disagreement rate of more than 20% in error assignment in this study, although this figure is not dissimilar from previously reported data.^25^

Most actions and programmes focused on maternal and neonatal mortality reduction rely on imprecise information on the actual causes of maternal mortality. WHO considers reduction of medical errors one of the key elements that defines quality of care, which in turn is fundamental to end preventable maternal mortality. Clinicians can only diagnose diseases they have thought about in the differential diagnostic process and for which they have been looking.^26^ It is resource-poor settings in which adequate diagnostic procedures are scarcer, and understaffing of the health system with restricted access to specialised clinicians common, where the comparison of autopsy findings with the clinical diagnosis could help improve clinical diagnostic performance by providing fundamental information.

## References

[1] WHO (2017). World Health Statistics 2017: monitoring health for the SDGs.

[2] Thaddeus S, Maine D (1994). Too far to walk: maternal mortality in context. Soc Sci Med.

[3] Shojania KG, Burton EC (2008). The vanishing non-forensic autopsy. N Engl J Med.

[4] Shojania KG, Burton EC, McDonald KM, Goldman L (2003). Changes in rates of autopsy-detected diagnostic errors over time: a systematic review. JAMA.

[5] Hinduja A, Gupta H, Dye D (2013). Autopsy proven causes of in hospital mortality in acute stroke. J Forensic Leg Med.

[6] Kuijpers CC, Fronczek J, van de Goot FR, Niessen HW, van Diest PJ, Jiwa M (2014). The value of autopsies in the era of high-tech medicine: discrepant findings persist. J Clin Pathol.

[7] Wittschieber D, Klauschen F, Kimmritz A-C (2012). Who is at risk for diagnostic discrepancies? Comparison of pre- and postmortal diagnoses in 1800 patients of 3 medical decades in East and West Berlin. PLoS One.

[8] Murray J, Sonnenberg P, Nelson G, Bester A, Shearer S, Glynn JR (2007). Cause of death and presence of respiratory disease at autopsy in an HIV-1 seroconversion cohort of southern African gold miners. AIDS.

[9] Cox JA, Lukande RL, Lucas S, Nelson AM, Van Marck E, Colebunders R (2010). Autopsy causes of death in HIV-positive individuals in sub-Saharan Africa and correlation with clinical diagnoses. AIDS Rev.

[10] Daramola AO, Elesha SO, Banjo AAF (2005). Medical audit of maternal deaths in the Lagos University Teaching Hospital, Nigeria. East Afr Med J.

[11] Ordi J, Ismail MR, Carrilho C (2009). Clinico-pathological discrepancies in the diagnosis of causes of maternal death in sub-Saharan Africa: retrospective analysis. PLoS Med.

[12] World Health Organization (2012). The WHO application of ICD-10 to deaths during pregnancy, childbirth and the puerperium: ICD-MM. WHO Libr.

[13] Hutchins GM, Berman JJ, Moore GW, Hanzlick R (1999). Practice guidelines for autopsy pathology: autopsy reporting. Arch Pathol Lab Med.

[14] Castillo P, Hurtado JC, Martínez MJ (2017). Validity of a minimally invasive autopsy for cause of death determination in maternal deaths in Mozambique: an observational study. PLoS Med.

[15] WHO (2016). International Classification of Diseases (ICD-10): international statistica classification of diseases and related health problems.

[16] Goldman L, Sayson R, Robbins S, Cohn LH, Bettmann M, Weisberg M (1983). The value of the autopsy in three medical eras. N Engl J Med.

[17] Battle RM, Pathak D, Humble CG (1987). Factors influencing discrepancies between premortem and postmortem diagnoses. JAMA.

[18] Landis JR, Koch GG (1977). The measurement of observer agreement for categorical data. Biometrics.

[19] Firth D (1993). Bias reduction of maximum likelihood estimates. Biometrika.

[20] Heinze G, Schemper M (2002). A solution to the problem of separation in logistic regression. Stat Med.

[21] Desale M, Thinkhamrop J, Lumbiganon P, Qazi S, Anderson J (2016). Ending preventable maternal and newborn deaths due to infection. Best Pract Res Clin Obstet Gynaecol.

[22] Schwanda-Burger S, Moch H, Muntwyler J, Salomon F (2012). Diagnostic errors in the new millennium: a follow-up autopsy study. Mod Pathol.

[23] Singh H, Schiff GD, Graber ML, Onakpoya I, Thompson MJ (2017). The global burden of diagnostic errors in primary care. BMJ Qual Saf.

[24] Saracci R (1991). Is necropsy a valid monitor of clinical diagnosis performance?. BMJ.

[25] Sonderegger-Iseli K, Burger S, Muntwyler J, Salomon F (2000). Diagnostic errors in three medical eras: a necropsy study. Lancet.

[26] Pelletier LL, Klutzow F, Lancaster H (1989). The autopsy: its role in the evaluation of patient care. J Gen Intern Med.

