# Supplementary material for: Quality of care and maternal mortality in a tertiary-level hospital in Mozambique: a retrospective study of clinicopathological discrepancies
Source: Lancet Glob Health. 2020 Jun 17;8(7):e965–72. doi: 10.1016/S2214-109X(20)30236-9 (PMC7303952; doi:10.1016/S2214-109X(20)30236-9)
Supplement: Supplementary appendix [file mmc1.pdf]

THE LANCET
Global Health

Supplementary appendix

This appendix formed part of the original submission and has been peer reviewed. We post it as supplied by the authors.

Supplement to: Menéndez C, Quintó L, Castillo P, et al. Quality of care and maternal mortality in a tertiary-level hospital in Mozambique: a retrospective study of clinicopathological discrepancies. *Lancet Glob Health* 2020; **8:** e965–72.

Appendix 1. The autopsy diagnosis and the first two clinical diagnoses for each particular case classified as major class I or II errors.

	Autopsy diagnosis	Clinical diagnosis (1)	Clinical diagnosis (2)
**Error**			
Class I	Cerebral cryptococcosis (HIV)	HIV disease resulting in encephalopathy	HIV disease
Class I	Miliary tuberculosis (HIV)	Eclampsia	Bacterial meningitis, not elsewhere classified
Class I	Puerperal sepsis	Acute pericarditis	Unspecified convulsions
Class I	Chronic obstructive pyelonephritis (HIV)	Respiratory failure, unspecified	HIV disease
Class I	Sepsis due to other Gram-negative organisms	HELLP syndrome	Premature separation of placenta, unspecified
Class I	Cryptococcosis, unspecified (HIV)	Pneumocystosis	Foetal death of unspecified cause
Class I	Miliary tuberculosis (HIV)	Fever of other and unknown origin	HIV disease
Class I	Streptococcal sepsis (HIV)	Plasmodium falciparum malaria	Spontaneous abortion
Class I	Puerperal sepsis	Eclampsia	Complications of anaesthesia during labour and delivery
Class I	Pneumonia, unspecified	Toxic effect of unspecified substance	Gastroenteritis and colitis of infectious origin
Class I	Plasmodium falciparum malaria, cerebral	Medical abortion	Gastroenteritis and colitis of infectious origin
Class I	Respiratory tuberculosis (HIV)	Bacterial pneumonia	..
Class I	Burkitt lymphoma (HIV)	Haemorrhage following abortion/ectopic pregnancy	..
Class I	Pneumonia due to staphylococcus (HIV)	Pre-eclampsia	Unspecified HIV disease
Class II	Pneumonia, unspecified (HIV)	Anaemia complicating pregnancy, childbirth and the puerperium	Genital tract infection following abortion/ectopic pregnancy
Class I	Cryptococcosis, unspecified (HIV)	Pulmonary oedema	HIV disease
Class I	Bacterial meningitis (HIV)	Encephalitis, unspecified	HIV disease
Class I	Puerperal sepsis	Cutaneous abscess, furuncle and carbuncle of trunk	Unspecified HIV disease
Class I	Miliary tuberculosis (HIV)	Gastroenteritis and colitis of infectious and unspecified origin	Intra-abdominal and pelvic swelling, mass and lump
Class II	Malignant neoplasm: Bronchus or lung	Pleural effusion, not elsewhere classified	Pre-eclampsia
Class I	Hepatic failure, unspecified	Anaemia complicating pregnancy, childbirth and the puerperium	Hypovolaemic shock
Class I	Pneumonia, unspecified (HIV)	Eclampsia	Disseminated intravascular coagulation
Class I	Pneumonia, organism unspecified	Genital tract infection following abortion/ectopic pregnancy	Adult respiratory distress syndrome
Class I	Pneumonia, organism unspecified (HIV)	Hypoglycaemia, unspecified	Tuberculosis
Class I	Pneumonia, organism unspecified	Anaemia, unspecified	Severe pre-eclampsia
Class I	Puerperal sepsis	Postpartum haemorrhage	Severe pre-eclampsia
Class I	Pneumonia, organism unspecified	Eclampsia	Pulmonary oedema
Class I	Oesophageal varices with bleeding	Premature separation of placenta [abruptio placentae]	Anaemia, unspecified
Class I	Sepsis, unspecified (HIV)	Tuberculous meningitis	HIV disease
Class I	Pneumonia, organism unspecified	Encephalopathy, unspecified	Pulmonary oedema
Class II	Puerperal sepsis	Peritonitis	Other sepsis
Class II	Unspecified pre-existing hypertension complicating pregnancy	Stroke, not specified as haemorrhage or infarction	Eclampsia
Class II	Bacterial pneumonia (HIV)	Adult respiratory distress syndrome	Encephalitis, myelitis and encephalomyelitis, unspecified
Class I	Pneumonia, organism unspecified (HIV)	Bacterial meningoencephalitis and meningomyelitis	HIV disease
Class II	Bacterial meningitis, unspecified (HIV)	Puerperal sepsis	..
